# Risk factors for predicting lateral lymph node metastasis of papillary thyroid carcinoma based on LASSO-logistic regression

**DOI:** 10.3389/fendo.2025.1642298

**Published:** 2025-09-22

**Authors:** Han Han, Lei Yang, WenJun Jia, Xiao Chen

**Affiliations:** ^1^ School of Nursing, Anhui University of Chinese Medicine, Hefei, China; ^2^ The Second Affiliated Hospital of Anhui Medical University, Hefei, China; ^3^ Thyroid surgery department, The Second Affiliated Hospital of Anhui Medical University, Hefei, China

**Keywords:** LASSO, papillary thyroid carcinoma, lateral lymph node metastasis, nomogram, logistic regression

## Abstract

**Background:**

Many clinicians are facing the dilemma about whether therapeutic lateral lymph node dissection (LLND) should be applied to treat papillary thyroid carcinoma (PTC) patients with suspicious lateral lymph node metastasis (LLNM). This research plans to construct a model to predict the risk of LLNM in PTC patients.

**Methods:**

389 PTC patients meeting the requirements were retrieved from the database of our hospital. The patients included were randomly divided into the training set (N1 = 244) and the validation set (N2 = 145). LASSO regression and logistic regression were used to screen the risk factors of LLNM. Receiver operating characteristic (ROC) curve, calibration curve and decision curve analysis (DCA) were used to measure the performance of the predictive model.

**Results:**

In this study, a predictive model for LLNM in PTC patients was established based on LASSO and logistic regression models. Nomogram was established for visualization. The analyses of the area under the curve (AUC), calibration curve and decision curve of the training set and validation set all performed well, indicating that the prediction model has net benefit and clinical practicability.

**Conclusions:**

Nomogram based on LASSO regression can predict the risk of preoperative LLNM in PTC patients. This model can assist doctors in formulating individualized postoperative follow-up plans for PTC patients.

## Introduction

In recent decades, the incidence of thyroid cancer has increased significantly. According to the Global Cancer Statistics 2020, thyroid cancer is now the ninth most common cancer worldwide (1). Among all malignant tumors, thyroid cancer exhibits the highest growth rate, with an annual increase of approximately 6% (2). Papillary thyroid carcinoma (PTC) is the most common primary thyroid malignancy, accounting for approximately 85–90% of all cases (3). Cervical lymph node metastasis is a frequent biological feature of PTC, occurring in 40–90% of cases (4). Cervical lymph node metastasis is classified into central lymph node metastasis (level VI) and lateral lymph node metastasis (LLNM) (levels II–V). According to the American Thyroid Association (ATA) guidelines, ipsilateral lobectomy is recommended for small, unifocal tumors without extrathyroidal extension or LNM, due to the indolent nature of PTC (5). In contrast, therapeutic lateral lymph node dissection (LLND) is advised for patients with clinically evident LLNM (5). LLNM is associated with a higher rate of regional recurrence than central LNM, leading to a poorer prognosis. Therefore, accurate preoperative identification of LLNM is critical for optimal staging, personalized treatment planning, and prognosis assessment.

International guidelines recommend therapeutic LLND for PTC patients with LLNM confirmed by preoperative imaging and pathology (6). Prophylactic LLND may be performed in patients with suspected but unconfirmed metastasis. However, LLND may constitute overtreatment in low-risk cases. Considering the complexity of neck anatomy, patients undergoing LLND carries risks such as bleeding, nerve injury, and lymphatic leakage (7). Hence, a systematic preoperative evaluation of LLNM is essential to determine the appropriate surgical strategy and extent.

Currently, suspicious lymph nodes are primarily assessed by ultrasound (US), computed tomography (CT), and fine-needle aspiration biopsy (FNAB) (8). FNAB is widely regarded as the gold standard for diagnosing LLNM (9). However, it is invasive and technically challenging when lymph nodes are small or located near critical anatomical structures. US is the first-line imaging modality for evaluating cervical lymph nodes (10). Its diagnostic accuracy is highly operator-dependent and can be limited by adjacent structures such as adipose tissue, the thyroid gland, and the trachea. The sensitivity of US for detecting LLNM ranges from only 20% to 40% (11). CT, which provides detailed anatomical visualization, is recommended as a complementary tool to US (12). Although CT is not the primary imaging modality for diagnosing PTC, it can offer advantages over US in identifying lymph node metastases in certain cases. For instance, Lee et al. found that CT outperforms US in detecting LLNM in patients with papillary thyroid microcarcinoma (13). Despite these advancements, accurately identifying LLNM remains clinically challenging. Improved prediction of LLNM risk would help clinicians and patients make more informed surgical decisions.

Several studies have developed LLNM risk prediction models for PTC (8, 14, 15). However, many rely solely on univariate and multivariate analyses, which are limited in addressing multicollinearity. LASSO regression, not widely applied in PTC research, can improve model performance by applying a penalty function to select relevant predictors. In this study, we integrate LASSO regression for variable selection with logistic regression for modeling and visualization, enabling more interpretable results. We aim to construct a new risk prediction model for LLNM in PTC patients using detailed preoperative examination results, thereby facilitating personalized clinical decision-making.

## Materials and methods

### Patients enrolled

This retrospective single-center study included PTC patients who underwent surgical treatment at the Second Affiliated Hospital of Anhui Medical University between January 1, 2017, and April 30, 2025. All selected patients underwent preoperative ultrasound and/or CT examinations, which revealed suspicious lateral cervical lymph nodes. All suspected lymph nodes were further evaluated using US-guided FNAB.

Inclusion criteria: (1) Patients who underwent thyroidectomy and LLND for the first time; (2) Histopathology confirmed PTC; (3) Suspicious imaging manifestations of lateral cervical lymph nodes; (4) Availability of complete clinical and pathological data. Exclusion criteria: (1) Prior thyroid surgery or treatment; (2) Pathologically confirmed LLNM preoperatively by FNAB; (3) Presence of distant metastases; (4) Coexisting malignancies.

**Figure 1:**
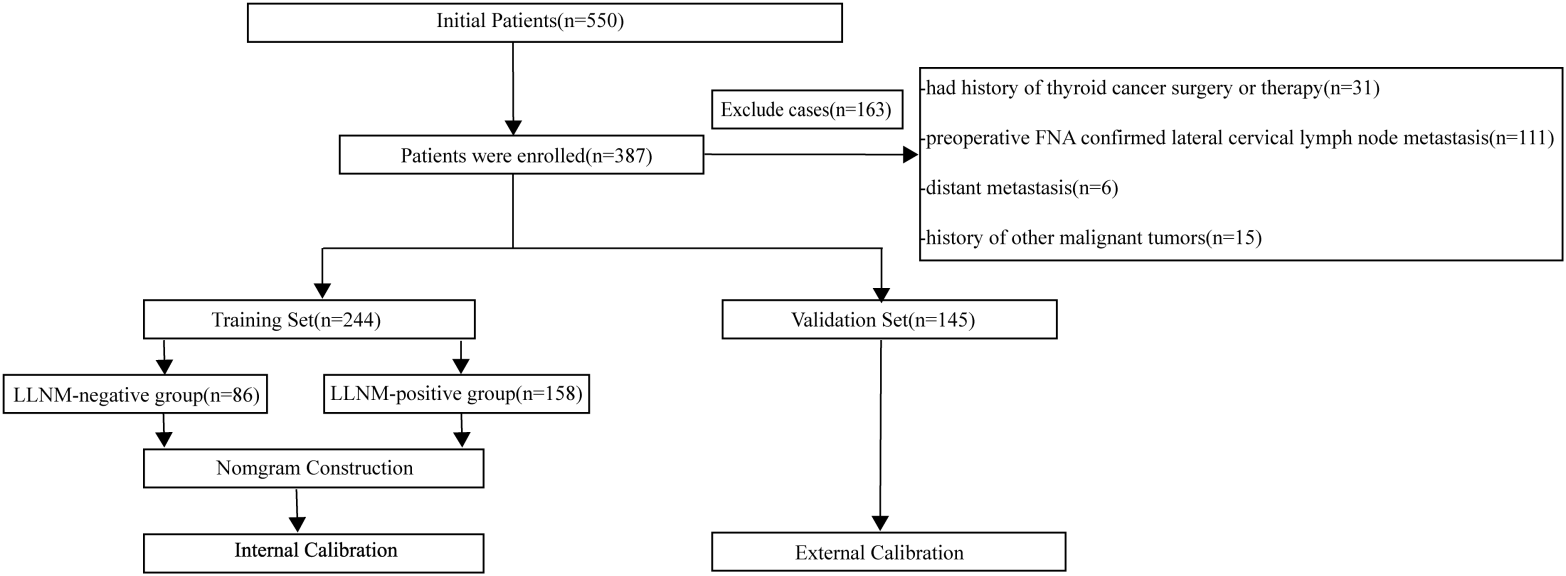
Flowchart of enrolled papillary thyroid carcinoma patients.

According to the empirical rule proposed by Peduzzi et al., logistic regression analysis requires at least 10 events per variable (EPV) to ensure model stability (16). Given that the final model may include 10 to 20 explanatory variables, a minimum of 100 PTC patients with LLNM was required. A total of 389 eligible patients were identified. The dataset was randomly divided into training and validation sets in an approximate 2:1 ratio. Patients were randomly divided into a training set (N1 = 244) and a validation set (N2 = 145). Based on postoperative pathological findings of lateral lymph nodes, patients in both the training and validation sets were categorized into LLNM-positive and LLNM-negative groups. A flowchart of patient inclusion is presented in **Figure 1**.

All clinical and demographic data were extracted from an electronic database containing records of PTC patients treated at our center. The Ethics Committee of the Second Affiliated Hospital of Anhui Medical University approved this study and waived the requirement for informed consent due to its retrospective design.

### Surgical indications

Surgical treatment for PTC included either total thyroidectomy or thyroid lobectomy with isthmus resection. Lobectomy with isthmus resection was indicated for solitary PTC confined to a single lobe with a tumor diameter ≤1 cm. Total thyroidectomy was performed when the primary lesion exceeded 4 cm in diameter, when bilateral multifocal disease was present, or when gross extrathyroidal invasion was evident intraoperatively. For patients with tumors measuring 1–4 cm, without obvious extrathyroidal invasion, and with contralateral thyroid nodules not suspected to be malignant, both surgical options were discussed. The risks and benefits of each approach were explained in detail, and the final surgical decision was made according to patient preference.

LLND was classified as either therapeutic or selective, depending on the clinical objective. Selective lateral cervical lymph node dissection is not routinely recommended for PTC, as occult metastasis does not significantly affect patient survival. Therefore, selective LLND is generally avoided in clinically node-negative (cN0) DTC patients at our institution. For patients with clinically node-positive (cN1) disease, the decision to perform LLND was based on imaging and FNAB:(1) If cytopathological examination confirmed malignancy in suspicious lymph nodes, LLND was performed. (2) If imaging suggested metastasis but cytopathology was inconclusive, a limited LLND (levels IIa, III, and IV) was initially performed, followed by intraoperative frozen section analysis to determine whether an extended dissection (levels II–V) was necessary. (3) If no suspicious LLNM was identified, LND was avoided.

### Imaging examination

All US examinations were performed with a Siemens S2000 system. A 6–15 MHz linear probe was used for conventional US. A 4–9 MHz probe for elastography. Two experienced sonographers (4 and 11 years of experience) independently evaluated the images. Disagreements were resolved by a third expert with over 20 years of experience. All reviewers were blinded to patient data and pathological results.

CT scans were conducted using a third-generation dual-source scanner (Somatom Definition, Siemens, Germany). Parameters included 90 kV and Sn 150 kV for tube A and B, respectively. Contrast-enhanced scans were performed with 60 mL of iodixanol injected at 3.5 mL/s. Arterial and venous phases were imaged at 25- and 50-second post-injection, respectively. Two radiologists (3 and 10 years of experience) reviewed the CT scans independently. Discrepancies were resolved by a third radiologist.

The gold standard for judging LLNM in this study is postoperative pathological diagnosis. During the operation, the surgeon removed the suspicious lymph nodes and sent them to the pathology department. Cervical lymph nodes were fixed with 20% buffered formalin, embedded in paraffin, and sectioned. All pathological specimens were reviewed and cross-examined under a microscope by two or more experienced pathologists.

### Data collection

The basic information, imaging results, laboratory items and pathological results of the patients were collected. Personal basic information includes age, gender and body mass index (BMI). According to clinical experience, the items determined to be included in the study include free triiodothyronine (FT3), free thyroxine (FT4), thyroid stimulating hormone (TSH), thyroglobulin (TG), thyroglobulin antibody (TGAb), thyroid peroxidase antibody (TPOAb), thyroid stimulating receptor antibody (TRAb), and carcinoembryonic antigen (CEA). All laboratory test results were recorded in the hospital’s Laboratory Information System (LIS) and retrieved via the Electronic Medical Record (EMR) system.

The imaging features of the tumor lesion include tumor size (defined as the maximum diameter of a single lesion or the largest diameter of multiple lesions), location, laterality (defined as having one or more malignant lesions in two glandular lobes), multifocality (defined as having two or more malignant lesions in one glandular lobe), location, echo,shape, margin, calcification, aspect ratio, tumor vascularity (the evaluation criteria are based on the Adler classification, which represents semi-quantitative classification), real-time elastography (RTE), and the relationship between the tumor and the capsule (Contact of capsule is defined as more than 25% of the tumor surface abutting or adjacent to the thyroid capsule. Disruption of capsule is defined as tumor invasion beyond the capsule).

Suspicious lymph nodes identified on ultrasound were also evaluated. Their sonographic features included size, multiregionality, corticomedullary differentiation, calcification, shape, and vascularity. Two radiologists with 3 and 10 years of experience in thyroid imaging independently assessed lymph node status using contrast-enhanced CT in a blinded manner. This assessment is referred to as the “CT-reported LN status”. Based on the National Comprehensive Cancer Network (NCCN) guidelines (6), metastatic lymph node features extracted from CT included shape, margin, calcification, and enhancement types.

In this study, the BRAF V600E mutation status was assessed in all patients through molecular analysis of FNAB specimens. All suspicious thyroid nodules underwent ultrasound-guided FNAB, performed by experienced ultrasound physicians. A minimum of three specimens was collected from each suspicious lesion. BRAF V600E mutation detection was conducted using real-time quantitative polymerase chain reaction (qPCR).

Clinical data, including laboratory test results and imaging reports, were collected through the hospital’s EMR and LIS systems. All preoperative assessments—including US, CT, and laboratory testing—were completed within one month prior to surgery. Postoperative pathological results were documented within one month following surgery.

### Statistical analysis

Statistical analyses were performed using SPSS software (version 23.0) and R software (version 4.4.1). Continuous variables were expressed as mean ± standard deviation. Continuous variables were tested for normality using the Shapiro–Wilk test. Variables with a normal distribution were analyzed using the t-test, while non-normally distributed variables were assessed using the Pearson chi-squared test. The chi-square test was used to evaluate associations between categorical variables. Due to the large number of multi-class variables, the group’s least absolute shrinkage and selection operator with 10-fold cross-validation were employed to identify the most predictive features in the training set (17). These features were incorporated into the LASSO regression and multivariate logistic regression model to establish a predictive nomogram. Model performance was assessed using receiver operating characteristic (ROC) curves in both the training and validation sets. Calibration curves and goodness-of-fit tests were used to evaluate model calibration and applicability in clinical practice. Decision curve analysis (DCA) was applied to assess the clinical utility of the model by estimating net benefit across different threshold probabilities (18). *P*-value<0.05 was considered statistically significant.

## Result

### Baseline patient characteristics

We evaluated 37 candidate variables and compared each detection metric of the training set and the validation set. The positive rates of LLNM in the training set and the validation set were 64.8% and 67.6% respectively. No significant difference was observed in the prevalence of LLNM between the two datasets. Furthermore, there were no significant differences in gender, age and BMI between the LLNM positive group and the negative group. **Table 1**; **Supplementary Table 1** show all the data of the studied patients.

**Table 1:** Characteristics of patients in the training set and validation set.

Characteristic	Training Set (n = 244)			Validation Set (n = 145)		
	LLNM(-) (n = 86)	LLNM(+) (n =158 )	P value	LLNM(-) (n =47 )	LLNM(+) (n = 98)	P value
Age (mean ± SD, range, years)	41.50±11.524	44.00±11.868	0.683	42.89 ± 12.513	44.05 ± 11.338	0.579
Sex (n, %)			0.803			0.132
Female	66 (76.7%)	119 (75.3%)		39 (83.2%)	70 (71.4%)	
Male	20 (23.3%)	39 (24.7%)		8 (17.0%)	28 (28.6%)	
BMI	24.304±3.726 (21.613-26.814)	24.219±3.348 (22.584-26.985)	0.497	23.088±3.769 (20.641-25.693)	22.44±3.202(20.602-25.205)	0.483
Benign Thyroid Disease(n, %)			0.671			0.089
No	16 (18.6%)	26 (16.5%)		9 (19.1%)	9 (9.2%)	
Yes	70 (81.4%)	132 (83.5%)		38 (80.9%)	89 (90.8%)	
Tumor Diameter(mm)	8 (6-11)	13 (9-20)	< 0.001	9 (7-12)	15 (10-20.75)	< 0.001
Laterality(n, %)			0.439			0.334
Unilateral	42 (48.8%)	69 (43.7%)		28 (59.6%)	50 (51.0%)	
Bilateral	44 (51.2%)	89 (56.3%)		19 (40.4%)	48 (49.0%)	
Multifocality (n, %)			< 0.001			0.003
No	62 (72.1%)	36 (22.8%)		23 (48.9%)	24 (24.5%)	
Yes	24 (27.9%)	122 (77.2%)		24 (51.1%)	74 (75.5%)	
Tumor Location(n, %)			< 0.001			0.264
Lower	36 (41.9%)	33 (20.9%)		13 (27.7%)	29 (29.6%)	
Middle	30 (34.9%)	50 (31.6%)		21 (44.7%)	31 (31.6%)	
Upper	20 (23.3%)	75 (47.5%)		13 (27.7%)	38 (38.8%)	
Tumor Echogenicity(n, %)			0.156			0.181
Hypoechogenicity	78 (90.7%)	146 (92.4%)		37 (78.7%)	81 (82.79%)	
Isoechoic	6 (7.0%)	12 (7.6%)		6 (12.8%)	15 (15.3%)	
Hyperechoic	2 (2.3%)	0 (0%)		4 (8.5%)	2 (2.0%)	
Tumor Shape(n, %)			0.275			0.119
Rule	40 (46.5%)	89 (56.3%)		24 (51.1%)	65 (66.3%)	
Near-regular	28 (32.6%)	46 (29.1%)		13 (27.7%)	23 (23.5%)	
Irregular	18 (20.9%)	23 (14.6%)		10 (21.3%)	10 (10.2%)	
Tumor Margin(n, %)			0.122			0.238
Clear	40 (46.5%)	95 (60.1%)		28 (59.6%)	67 (68.4%)	
Near-clear	32 (37.2%)	43 (27.2%)		16 (34.0%)	21 (21.4%)	
Unclear	14 (16.3%)	20 (12.7%)		3 (6.4%)	10 (10.2%)	
Tumor Calcification(n, %)			0.584			0.02
None	22 (25.6%)	33 (20.9%)		10 (21.3%)	20 (20.4%)	
Coarse Calcification	46 (53.5%)	95 (60.1%)		18 (38.3%)	63 (64.3%)	
Fine Calcification	18 (20.9%)	30 (19.0%)		19 (40.4%)	15 (45.3%)	
Tumor Length(n, %)			0.964			0.585
Width ratio ≤1	22 (25.6%)	40 (25.3%)		18 (38.3%)	33 (33.7%)	
Width ratio >1	64 (74.4%)	118 (74.7%)		29 (61.7%)	65 (66.3%)	
Tumor Vascularity(n, %)			0.063			0.716
No Blood Flow Signal	20 (23.3%)	37 (23.4%)		7 (14.9%)	15 (15.3%)	
Punctate Blood Flow Signal	44 (51.2%)	59 (37.3%)		19 (40.4%)	33 (33.7%)	
Enriched Blood Flow Signal	22 (25.6%)	62 (39.2%)		21 (44.7%)	50 (51.0%)	
RTE(n, %)			0.315			0.174
2	1 (1.2%)	2 (1.3%)		1 (2.1%)	2 (2.0%)	
3	56 (65.1%)	118 (74.7%)		20 (42.6%)	55 (56.1%)	
4	20 (23.3%)	22 (13.9%)		20 (42.6%)	24 (24.5%)	
5	9 (10.5%)	16 (10.1%)		6 (12.8%)	17 (17.3%)	
Invasion (n, %)			< 0.001			0.002
Continuity of Capsule	48 (55.8%)	44 (27.8%)		25 (53.2%)	25 (25.5%)	
Contact of Capsule	33 (38.4%)	75 (47.5%)		18 (38.3%)	49 (50.0%)	
Disruption of Capsule	5 (5.8%)	39 (24.7%)		7(8.5%)	24 (24.5%)	
BRAFV600E Mutation(n, %)			0.308			0.078
No	15 (17.4%)	20 (12.7%)		13 (27.7%)	15 (15.3%)	
Yes	71 (82.6%)	138 (87.3%)		34 (72.3%)	83 (84.7%)	
Maximum Diameter of Suspicious LN on US (mm)	9±3.551 (7-9)	13±6.560 (10-17)	< 0.001	10±4.180 (8-11)	15±10.504 (13-20)	< 0.001
Multiregionality of Suspicious LN on US (n, %)			< 0.001			0.010
No	72 (83.7%)	97 (61.4%)		38 (80.9%)	58 (59.2%)	
Yes	14 (16.3%)	61 (38.6%)		9 (19.1%)	40 (40.8%)	
Shape of Suspicious LN on US (n, %)			0.128			0.680
Rule	72 (83.7%)	119 (75.3%)		36 (76.6%)	78 (79.6%)	
Regular	14 (16.3%)	39 (24.7%)		11 (23.4%)	20 (20.4%)	
Corticomedullary Differentiation of Suspicious LN on US (n, %)			0.001			0.002
Clear	36 (41.9%)	69 (43.7%)		18 (38.3%)	40 (40.8%)	
Near-clear	38 (44.2%)	39 (24.7%)		23 (48.9%)	23 (23.5%)	
Unclear	12 (14.0%)	50 (31.6%)		6 (12.8%)	35 (35.7%)	
Calcification of Suspicious LN on US (n, %)			0.975			0.648
None	41 (47.7%)	75 (47.5%)		32 (68.1%)	60 (61.2%)	
Microcalcification	45 (52.3%)	83 (52.5%)		15 (31.9%)	38 (38.8%)	
Blood Flow Signals of Suspicious LN on US (n, %)			< 0.001			< 0.001
Lymphoid Portal	42 (48.8%)	55 (34.8%)		23 (48.9%)	29 (29.6%)	
Punctate	36 (41.9%)	31 (19.6%)		18 (38.3%)	22 (22.4%)	
Mixed	4 (4.7%)	39 (24.7%)		3 (6.4%)	26 (26.5%)	
Enriched	4 (4.7%)	33 (20.9%)		3 (6.4%)	21 (21.4%)	
CT reported LN Status (n, %)			< 0.001			< 0.001
Negative	76 (88.4%)	50 (31.6%)		42 (89.4%)	32 (32.7%)	
Positive	10 (11.6%)	108 (68.4%)		5 (10.6%)	66 (67.3%)	
Shape of LN on CT (n, %)			0.198			0.746
Rule	68 (79.1%)	113 (71.5%)		26 (55.3%)	57 (58.2%)	
Regular	18 (20.9%)	45 (28.5%)		21 (44.7%)	41 (41.8%)	
Margin of LN on CT (n, %)			0.010			0.030
Clear	68 (79.1%)	97 (61.4%)		38 (80.9%)	58 (59.2%)	
Unclear	18 (20.9%)	56 (35.4%)		9 (19.1%)	38 (38.3%)	
Organizational infringement	0 (0%)	5 (3.2%)		0 (0%)	2 (2.0%)	
Calcification of LN on CT (n, %)			< 0.001			< 0.001
No	57 (66.3%)	66 (41.8%)		34 (72.3%)	38 (38.7%)	
Yes	29 (33.7%)	92 (58.2%)		13 (27.7%)	60 (61.2%)	
Enhanced Types of LN on CT (n, %)			< 0.001			< 0.001
No	58 (67.4%)	10 (6.3%)		31 (66.0%)	6 (6.1%)	
Uneven Reinforcement	16 (18.6%)	99 (62.7%)		10 (21.3%)	65 (66.3%)	
Obvious Reinforcement	12 (14.0%)	49 ( 31.0%)		6 (12.8%)	27 (27.6%)	
FT3	5.6778±2.256 (4.05-7.6195)	5.5482±2.284 (3.6616-7.2792)	0.333	5.6778±2.161 (4.6163-6.6742)	5.61±2.305 (3.6715-7.3274)	0.542
FT4	14.758±5.568 (10.915-18.997)	15.019±6.030 (9.8032-19.919)	0.728	14.662±5.537 (10.923-18.489)	13.975±5.929 (9.0163-18.79)	0.737
TSH	4.8027±2.570 (3.3412-6.6564)	4.8049±2.952 (2.1276-7.5479)	0.495	5.0728±2.683 (3.458-7.3076)	4.5682±2.882 (2.2422-6.952)	0.184
TRAb	4.2795±2.471 (1.6773-6.1148)	3.8643±2.210 (2.0252-5.6355)	0.669	3.62±2.548 (1.4663- 6.0867)	3.9661±2.224 (2.0584-5.6911)	0.811
TG	15.863±25.415 (9.2017-26.72)	24.042±55.277 (13.135-59.153)	< 0.001	17.329±29.830 (9.9077-26.968)	22.916±46.842 (12.383-34.986)	0.037
TBG	6.2605±3.554 (4.2182-8.4364)	6.006±2.276 (3.7591-7.7725)	0.285	6.260±4.241 (4.3157- 8.5533)	6.2183±2.256 (3.8377-7.7195)	0.474
TGAb	5.6904±2.957 (4.0464-8.5256)	6.1699±2.354 (4.2003-8.2911)	0.804	5.682±3.352 (4.4405-8.6721)	6.404±2.443 (4.3728-8.5617)	0.914
TPOAb	20.517±82.316 (15.782-26.323)	20.503±47.854 (14.575-25.664)	0.481	21.862±88.98. (15.88-26.216)	19.54±54.922 (14.489-25.496)	0.337
CEA	3.8298±2.095 (2.3472-6.5224)	4.3607±2.062 (2.7639-6.3971)	0.204	3.8298±2.221 (2.278-6.5616)	4.3862±2.010 (2.7185-6.1583)	0.680

LN, lymph node; RTE, real-time elastography; FT3, free triiodothyronine; FT4, free thyroxine; TSH, thyroid stimulating hormone; TG, thyroglobulin; TGAb, thyroglobulin antibody; TPOAb, thyroid peroxidase antibody; TRAb, thyroid stimulating receptor antibody; CEA, carcinoembryonic antigen.

### Construction of the predictive model and nomogram

**Figure 2:**
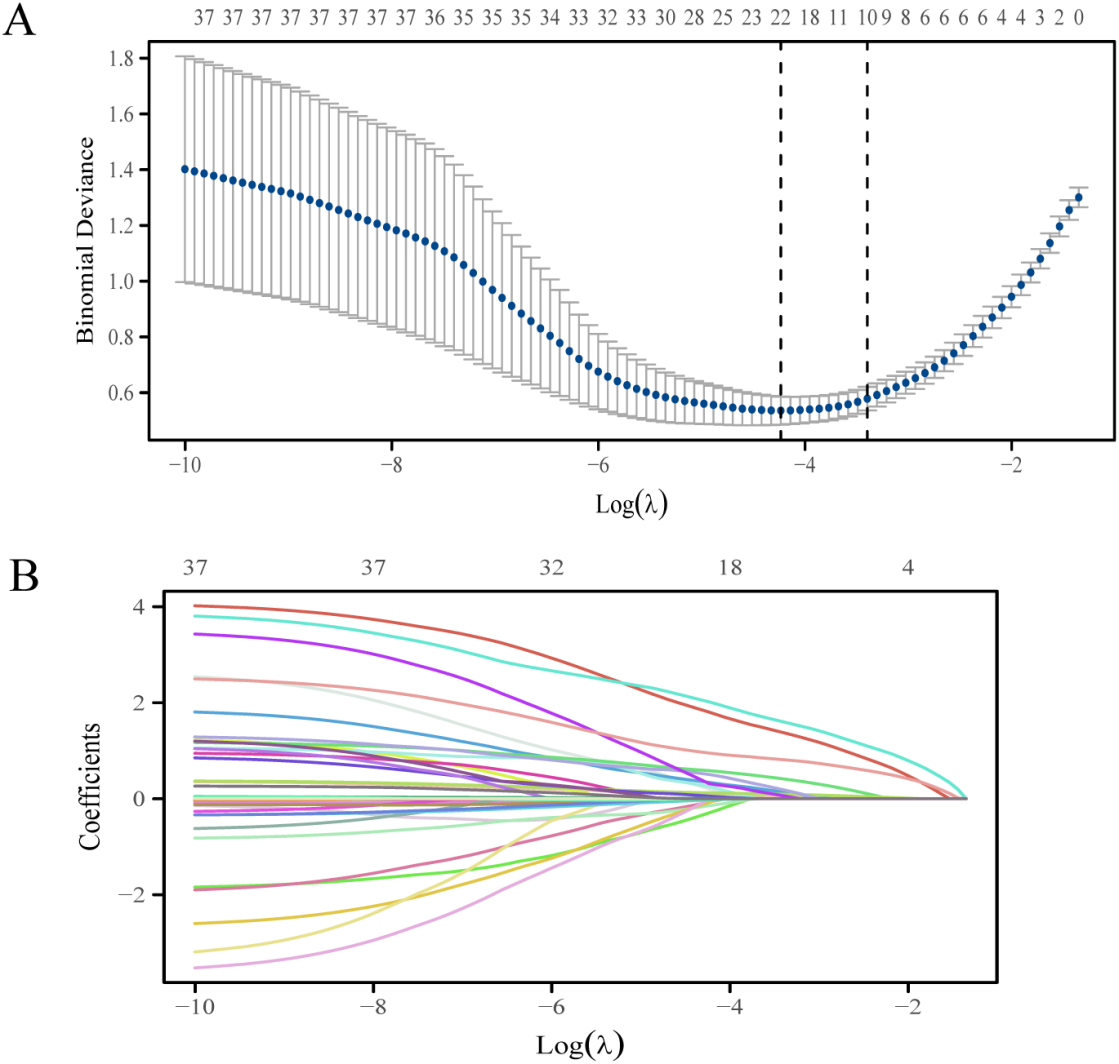
The LASSO regression variable screening process. **(A)** Optimization parameters of the LASSO model were selected using tenfold cross-validation. The two dotted vertical lines were drawn at the optimal scores by lambda.min and lambda.1se. **(B)** LASSO coefficient profiles of the 37 variables.

**Figure 2** shows the variation of the coefficients of each variable in LASSO regression with the penalty parameter (λ). The vertical dotted line marks the optimal λ value determined through 10-fold cross-validation. Under the optimal λ (lambda.1se), LASSO regression screened out 10 variables with non-zero coefficients, including tumor diameter, multifocality, tumor location, invasion, maximum diameter of suspicious LN on US, multiregionality of suspicious LN on US, CT reported LN status, calcification of LN on CT, enhanced types of LN on CT and TG (**Supplementary Table 2**). Logistic regression analysis showed that multifocality and TG were risk predictors in the prediction model (**Table 2**).

**Table 2:** Prediction factors and coefficients of the prediction model based on multivariate regression.

Characteristics	Coefficient	OR(95% CI)	P value
(Intercept)	-6.950		
Tumor Diameter	0.201	1.223 (1.083 – 1.381)	0.001
Multifocality			
No		Reference	
Yes	3.117	22.571 (4.978 – 102.349)	< 0.001
Tumor Location			
Middle		Reference	
Lower	-0.242	0.785 (0.145 – 4.253)	0.779
Upper	2.346	10.440 (1.740 – 62.647)	0.01
Invasion			
Contact of Capsule		Reference	
Disruption of Capsule	2.805	16.533 (1.803 – 151.572)	0.013
Continuity of Capsule	-0.102	0.903 (0.212 – 3.846)	0.891
Maximum Diameter of Suspicious LN on US	0.174	1.190 (1.049 – 1.350)	0.007
Multiregionality of Suspicious LN on US			
No		Reference	
Yes	1.161	3.194 (0.671 – 15.198)	0.145
CT reported LN Status			
Negative		Reference	
Positive	2.624	13.788 (3.269 – 58.151)	< 0.001
Enhanced Types of LN on CT			
Uneven Reinforcement	-4.316	Reference	
No	-1.150	0.013 (0.002 – 0.089)	< 0.001
Obvious Reinforcement		0.317 (0.070 – 1.428)	0.135
TG	0.043	1.044 (1.009 – 1.079)	0.012

**Figure 3:**
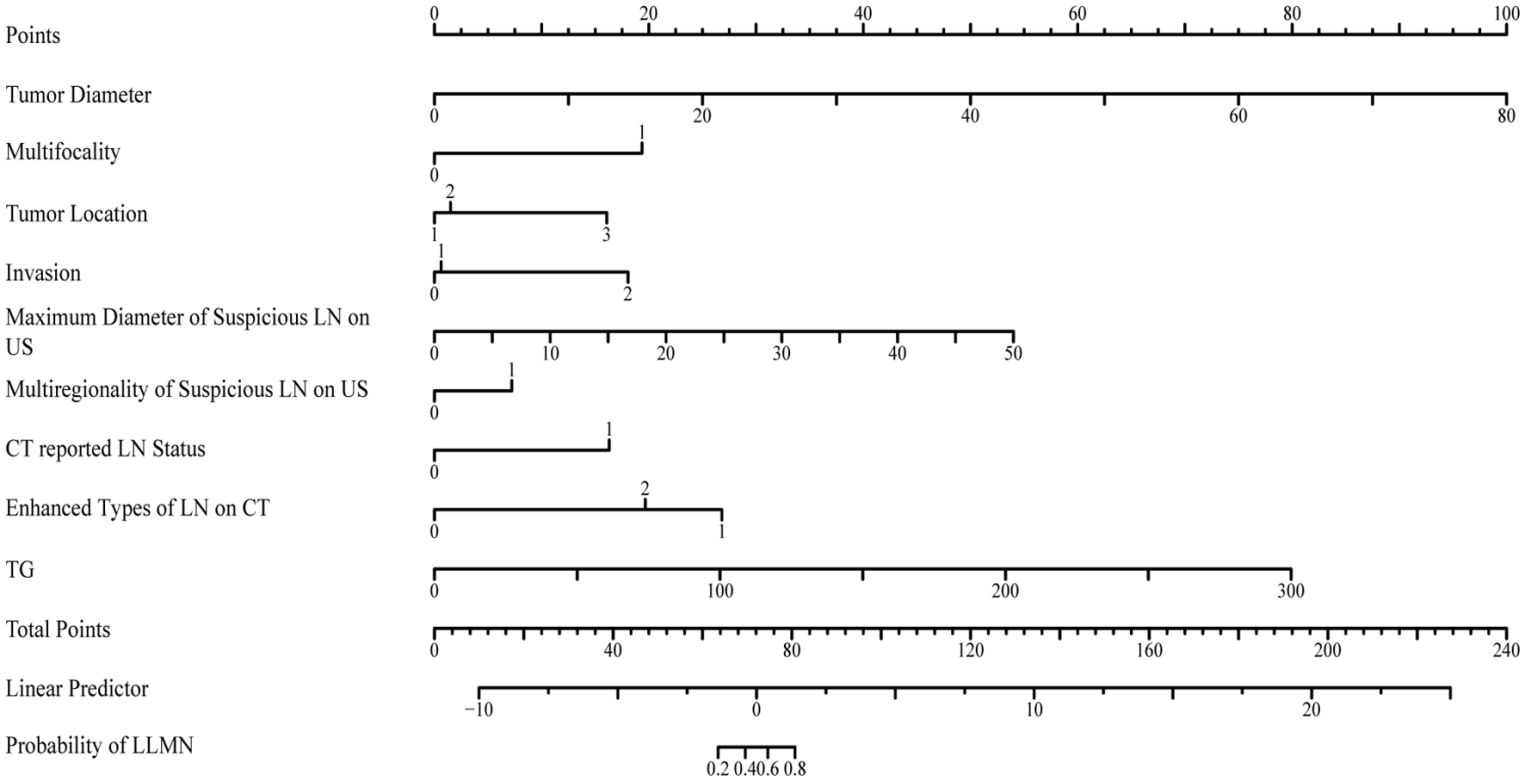
The nomogram for predicting the lateral lymph node metastasis probability in papillary thyroid carcinoma patients.

The risk predictors obtained through LASSO and logistic regression were visualized in nomogram (**Figure 3**). In the constructed nomogram, each predictor variable corresponds to a scoring axis. Based on an individual patient’s characteristics, scores are read from each variable’s axis and then summed to obtain a total score. The total score is located on the “Total Score” axis and projected vertically downward to the “LLNM Risk” axis to estimate the probability of LLNM.

### Performance of LLNM risk nomogram

**Figure 4:**
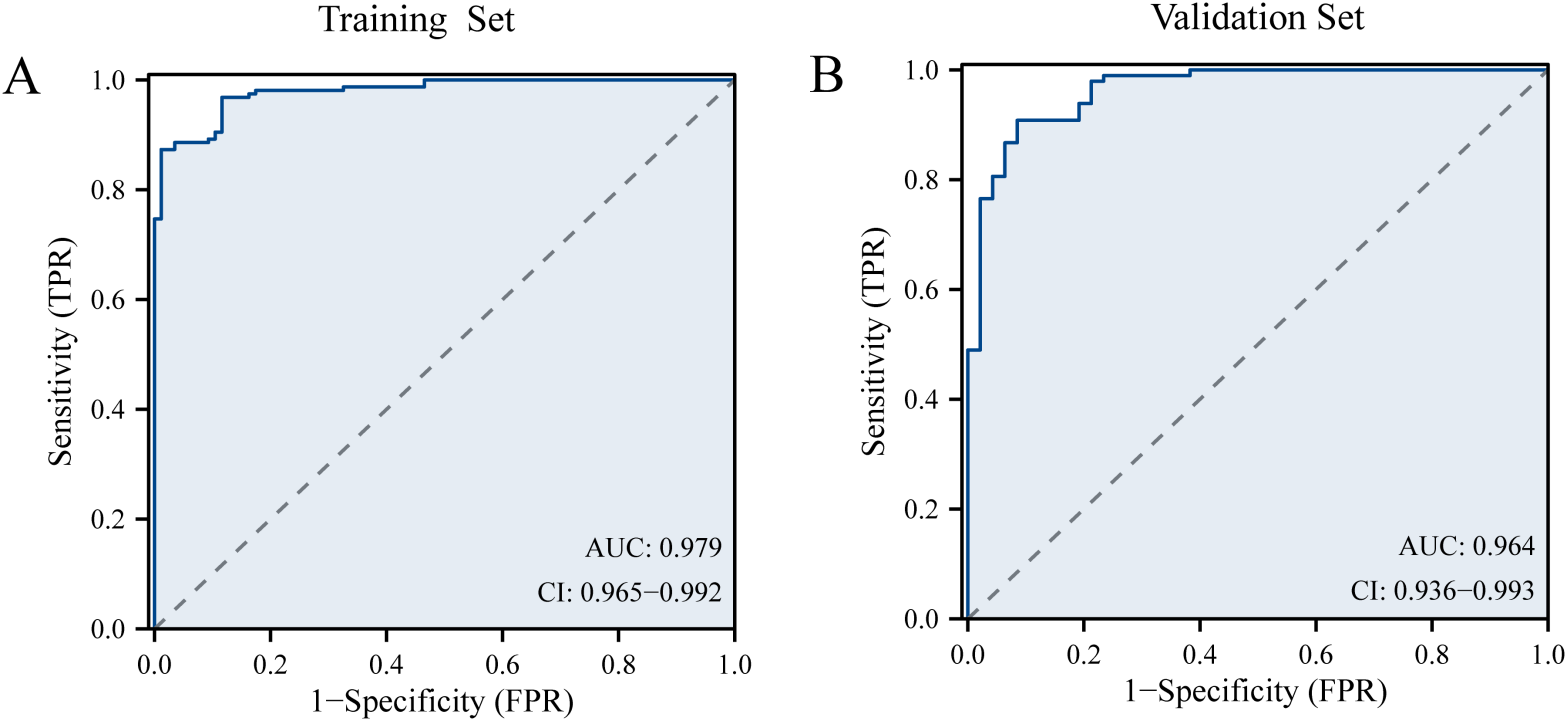
**(A)** The receiver operating characteristic curve of prediction model in the training set. **(B)** The receiver operating characteristic curve of prediction model in the validation set.

The discriminative performance of the model was assessed using the concordance index (C-index) and the area under the curve (AUC). Internal validation was conducted using the validation set. The C-index of the training set was 0.914. The AUC of the training set was 0.979 (95%CI: 0.965-0.992), with a sensitivity of 0.873 and a specificity of 0.988 (**Figure 4**). In the validation set, the C-index and AUC were 0.910 and 0.964 (95% CI, 0.936-0.993), respectively. The LLNM risk nomogram demonstrated strong predictive ability among PTC patients.

### Decision curve analysis and clinical impact curve

**Figure 5:**
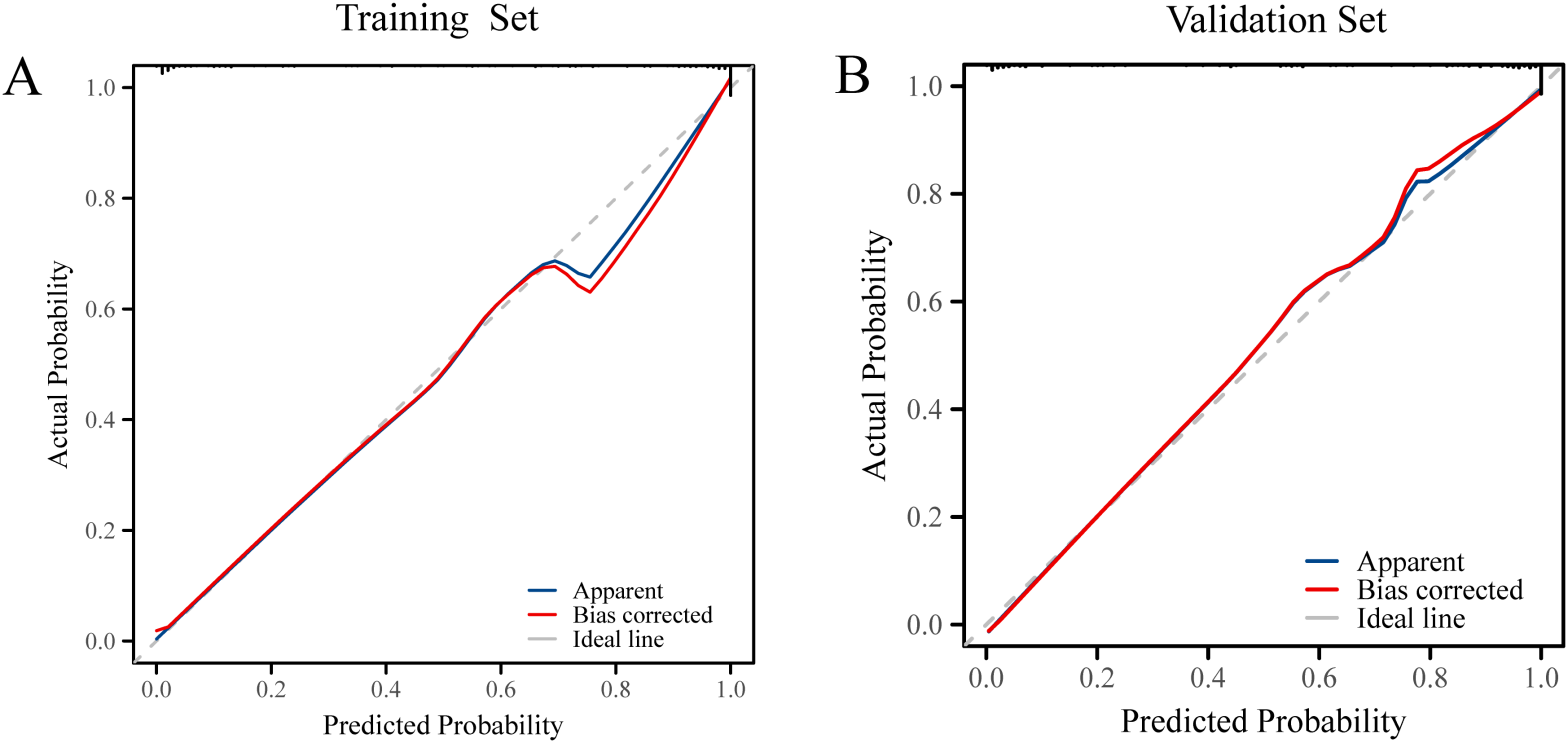
**(A)** Calibration curves of the prediction model in the training set. **(B)** Calibration curves of the prediction model in the validation set.

Model calibration was assessed using calibration curves, which demonstrated good agreement between predicted and observed probabilities in both the training and validation sets (**Figure 5**). The Hosmer–Lemeshow test yielded *P*-values > 0.05 for both datasets, indicating adequate model fit.

**Figure 6:**
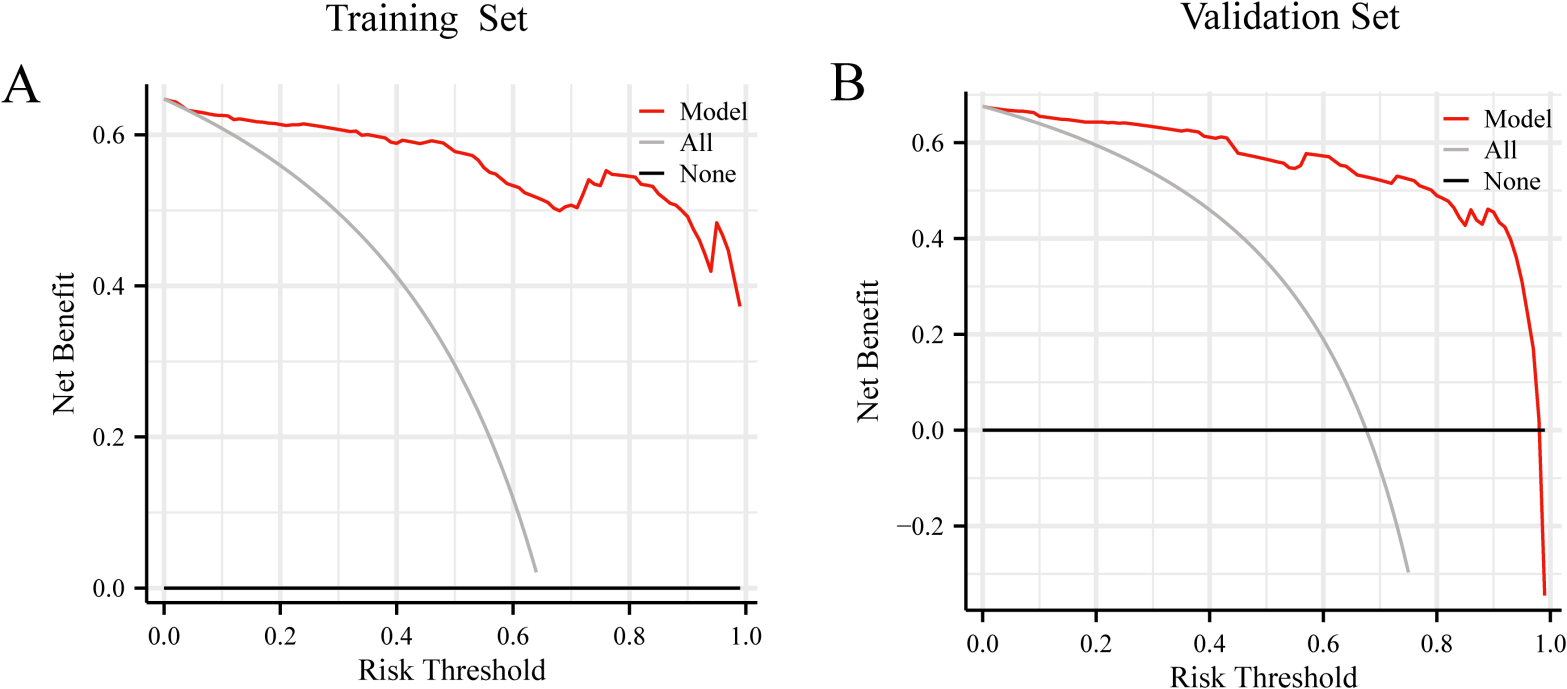
**(A)** Decision curve analysis of prediction model in the training set. **(B)** Decision curve analysis of prediction model in the validation set.

DCA was performed for the nomogram in both the training and validation sets. **Figure 6** presents the decision curve results, showing that the nomogram provides a positive net benefit when the threshold probability ranges from 37% to 65%. This suggests that the prediction model offers clinically meaningful guidance in this probability range. When the predicted LLNM probability ranges between 37% and 65%, the model recommends consideration of LLND, thereby enhancing the precision and effectiveness of surgical decision-making.

## Discussion

In recent years, the incidence of PTC has steadily increased (1). Although the prognosis of PTC is generally favorable, the rate of LLNM remains relatively high (2). Conventional wisdom suggests that the presence of LLNM in PTC patients is often associated with poor prognosis and an increased risk of local recurrence (3). Recent studies suggest that many intermediate-risk patients have favorable prognoses, supporting the trend toward less extensive surgical intervention. For instance, a study involving 946 patients with unilateral PTC and ipsilateral clinically apparent lateral neck metastases (cN1b) reported similar recurrence-free survival (RFS) outcomes between those who underwent lobectomy and those who underwent total thyroidectomy (19). Similarly, a 2025 study published in JAMA Otolaryngology demonstrated comparable survival and recurrence outcomes in intermediate-risk cN1b PTC patients treated with either lobectomy plus ipsilateral neck dissection or total thyroidectomy plus ipsilateral dissection (20). The 2021 ATA guidelines explicitly discourage prophylactic LLND, emphasizing that LLND should be performed for therapeutic rather than preventive purposes (5). Prophylactic LLND in patients with only suspected LLNM has not been shown to improve prognosis and may result in postoperative complications and visible cervical scarring, adversely affecting patients’ quality of life (21). Therefore, accurate preoperative assessment of cervical lymph node status is essential for selecting an appropriate treatment strategy. Enhancing the predictive accuracy for LLNM using current technologies has become a key issue in clinical practice. The nomogram developed in this study aims to identify patients truly at high risk for LLNM, thereby achieving a balance between minimizing unnecessary surgical intervention and reducing the risk of recurrence. Numerous studies have explored the risk factors associated with LLNM, but the findings remain inconsistent (22, 23). Liang et al. identified several key risk factors contributing to the development of LLNM in thyroid cancer, including central lymph node metastasis, extrathyroidal extension, multifocality, male sex, tumor location in the upper thyroid pole, tumor volume, lymph vascular invasion, and bilateral tumor presence (22). Similarly, Lai et al. reported that tumor size, location, microcalcification of lymph nodes, lymph node size, and patient age were significant predictors of LLNM (23). Although prophylactic LLND is not routinely recommended, preoperative imaging may reveal suspected lateral lymph node metastases, necessitating surgical intervention. In our cohort, patients in the LLNM-negative group showed suspicious metastases on imaging, but no metastases were confirmed by postoperative pathology. This finding suggests a potential false-positive rate in preoperative imaging, underscoring the critical importance of accurate preoperative assessment in guiding surgical decision-making. In this study, we identified tumor diameter, multifocality, tumor location, invasion, maximum diameter of suspicious LN on US, multiregionality suspicious LN on US, CT reported LN status, calcification of LN on CT, enhanced types of LN on CT and TG as significant risk factors for LLNM in PTC. All these variables were incorporated into the clinical prediction model. Nomogram was used to estimate the individual risk of LLNM in PTC patients, thereby helping clinicians determine whether LLND is needed.

In our study, the incidence rates of LLNM were 64.8% and 67.6% in the two groups, which was higher than previous reports (35.2-44.5%) (24, 25). This study only included PTC patients whose preoperative examinations highly suspected LLNM, which might explain the higher LLNM rate observed in our study than in previous studies. Previous studies have found that tumor size larger than 1.0 cm is significantly associated with LLNM (22). Larger tumor size is not only associated with a higher risk of LLNM (26), but also an independent predictor of LLNM recurrence (27). In our research cohort, tumor size was associated with LLNM in PTC.

Studies have shown that both bilateral and multifocal tumor characteristics are independent risk factors for LLNM in thyroid cancer (28). Liu et al. demonstrated a significant association between unilateral multifocality and LLNM, suggesting that multifocality increases the likelihood of lymphatic metastasis in thyroid cancer (29). In our study, we confirmed that tumor multifocality was an independent risk factor for LLNM, whereas laterality was not.

Previous studies have demonstrated that the location of thyroid cancer is closely associated with LLNM (30). Research by Jee indicates that nodules located in the upper pole or in the upper to middle third of the thyroid gland are significantly more prone to LLNM compared to those in the middle pole (31). This increased risk is primarily due to the similarity between the lymphatic and venous drainage systems of the thyroid, which collect lymph from the upper and middle poles (32). Lai et al. also reported that tumors located in the upper pole exhibit a significantly higher risk of LLNM (23). Therefore, we recommend that the preoperative assessment of thyroid nodules in the upper and middle poles should include careful assessment of the lateral cervical lymph nodes.

Extraglandular and capsular invasion of thyroid cancer are indicative of increased tumor aggressiveness. Huang et al. identified extraglandular invasion as a strong predictor of LLNM (33). Similarly, Yang et al. found that patients with capsular infiltration had a higher risk of LLNM than those without (34). Additional studies have shown that the proximity of the tumor to the thyroid capsule correlates with lateral cervical lymph node metastasis (35). Specifically, the closer a nodule is to the capsule, the greater the likelihood of LLNM.

Metastatic lymph nodes typically exhibit features such as calcification, cystic necrosis, hyperechogenicity, round shape, peripheral or mixed vascularity, and absence of an echogenic hilum. US is the primary non-invasive imaging modality for the preoperative evaluation of lymph node status. Accordingly, in this study, we focused on the US characteristics of cervical lymph nodes to assess their predictive value for LLNM in PTC. The size of lymph nodes is a critical factor in assessing lymph node status. Various studies have proposed different thresholds for defining suspicious lymph nodes. Generally, a diameter of 10 mm is considered the standard cutoff for identifying potential metastasis (36). Mack et al. found that spherical lymph nodes larger than 10 mm are more likely to indicate malignancy (37). Additionally, studies have shown that the likelihood of metastasis is 25.46 times higher in regions with multiple suspicious lymph nodes compared to regions with a single suspicious node (8). In our study, both the maximum diameter and multiregionality of suspicious lymph nodes on US were significantly associated with LLNM in PTC patients.

CT has demonstrated advantages in the evaluation of LLNM, as it effectively visualizes lymph nodes and delineates their spatial relationships with adjacent blood vessels (38). Morphological CT features—such as lymph node size >10 mm, irregular shape, ill-defined margins, calcification, and heterogeneous enhancement—are commonly used as predictive indicators of metastasis (39). Liu et al. reported a correlation between the degree of lymph node enhancement and metastatic involvement (40). Zhao et al. highlighted shape, margin, calcification, and cystic changes as key morphological characteristics of metastatic lymph nodes (41). In our study, we found significant differences in calcification and enhancement patterns between suspicious lymph nodes, but no differences in shape or margin between LLNM-negative and LLNM-positive groups. We propose that this discrepancy may be attributed to the subjectivity and variability inherent in morphological assessments.

TG, secreted by thyroid follicular epithelial cells, serves as a precursor for the synthesis of thyroid hormones. The American Thyroid Association recommends routine monitoring of serum TG levels in patients undergoing thyroid cancer surgery (5). Postoperative serum TG levels, particularly following thyroidectomy and radioactive iodine ablation are commonly used as indicators of residual thyroid tissue or metastatic disease (42). Elevated preoperative TG levels may also raise suspicion of cervical lymph node involvement (43). We found that elevated serum TG was associated with an increased risk of LLNM in PTC.

In the ongoing debate over LLND in PTC, our model offers a valuable reference for selecting personalized surgical approaches through non-invasive preoperative assessment. While numerous predictive models for diagnosing LLNM have been developed, they primarily rely on logistic regression for variable selection and model construction (8, 14, 15). These models often suffer from limitations such as small sample sizes, incomplete evaluation methodologies, and a lack of internal or external validation.

To address these limitations, we constructed a personalized and quantitative nomogram to predict the probability of LLNM in PTC patients using LASSO regression and multivariate logistic regression analysis. The study cohort was randomly divided into a training set for model development and a validation set for internal validation. ROC curves, calibration plots, and DCA were employed to assess the model’s accuracy and robustness. Although the model demonstrated excellent discrimination (AUC > 0.95) in both the training and validation cohorts, current evidence does not support its use in expanding surgical indications. Studies have shown that while prophylactic LLND can detect occult metastases in approximately 40% of patients, it does not improve oncologic outcomes (19, 20). Therefore, even when the model predicts a high probability of LLNM, central lymph node dissection (CLND) remains the standard surgical approach, unless cN1b disease is confirmed through preoperative fine-needle aspiration or intraoperative frozen section analysis (44). The primary value of this model lies in its utility for postoperative risk stratification. For patients with a high predicted probability (total score >75; LLNM risk >65%), more intensive postoperative surveillance—such as neck ultrasound every 3–6 months and dynamic serum Tg monitoring—is recommended to facilitate early detection of late lateral neck recurrence. Conversely, for patients with a low predicted probability (total score <60; LLNM risk <20%), extended follow-up intervals may be appropriate, thereby reducing unnecessary imaging and lowering the healthcare burden.

However, our study has several limitations. First, the nomogram was developed using data from a single-center study, which may introduce selection bias. While internal validation results are promising, external validation is essential to confirm the model’s generalizability. Future studies will incorporate data from multiple centers to evaluate its applicability across diverse patient populations. Second, as this study is based on a retrospective cohort, the model requires further prospective validation prior to clinical application. Finally, some imaging features are subjectively assessed and may vary between observers. To minimize interobserver variability, a third radiologist was consulted during final evaluations.

## Conclusion

In summary, tumor diameter, multifocality, tumor location, invasion, maximum diameter of suspicious LN on US, multiregionality suspicious LN on US, CT reported LN status, calcification of LN on CT, enhanced types of LN on CT and TG are risk factors for predicting LLNM in PTC patients. The nomogram constructed from these variables demonstrated strong predictive performance and holds potential as a practical tool for individualized LLNM risk assessment, aiding clinicians in risk stratification and postoperative follow-up planning.

## Data Availability

The raw data supporting the conclusions of this article will be made available by the authors, without undue reservation.
