# Supplementary material for: Risk factors for predicting lateral lymph node metastasis of papillary thyroid carcinoma based on LASSO-logistic regression
Source: Front Endocrinol (Lausanne). 2025 Sep 22;16:1642298. doi: 10.3389/fendo.2025.1642298 (PMC12497606; doi:10.3389/fendo.2025.1642298)
Supplement: Supplementary file 1 [file Table1.doc]

|  | **Supplementary Table 1** Characteristics of patients in the training set and validation set. | | | | | | |
| --- | --- | --- | --- | --- | --- | --- | --- |
| **Characteristic** | **Training Set (n = 244)** | | |  | **Validation Set (n = 145)** | | |
|  | **LLNM(-) (n = 86)** | **LLNM(+) (n =158 )** | **P value** |  | **LLNM(-) (n =47 )** | **LLNM(+) (n = 98)** | **P value** |
| Types of thyroid surgery |  |  | 0.425 |  |  |  | 0.718 |
| Lobectomy | 29 (33.7%) | 60 (38.0%) |  |  | 18 (38.3%) | 35 (35.7%) |  |
| TT | 57 (66.3%) | 98 (62.0%) |  |  | 29 (61.7%) | 63 (64.3%) |  |
| CLNM |  |  | 0.118 |  |  |  | 0.302 |
| No | 23 (26.7%) | 57 (36.1%) |  |  | 15 (31.9%) | 40 (40.8%) |  |
| Yes | 63 (73.3%) | 101 (63.9%) |  |  | 32 (68.1%) | 58 (59.2%) |  |

TT, total thyroidectomy; CLNM, central lymph node metastasis.
