# Supplementary material for: Risk factors for predicting lateral lymph node metastasis of papillary thyroid carcinoma based on LASSO-logistic regression
Source: Front Endocrinol (Lausanne). 2025 Sep 22;16:1642298. doi: 10.3389/fendo.2025.1642298 (PMC12497606; doi:10.3389/fendo.2025.1642298)
Supplement: Supplementary file 2 [file Table2.doc]

**Supplementary Table 2 The penalty parameter (λ) of variables with non-zero coefficients.**

| Variable | Lambda.min |
| --- | --- |
| (Intercept) | -5.33941909 |
| Age | 0 |
| Sex | 0 |
| BMI | 0 |
| Benign Thyroid Disease | -0.29406512 |
| Tumor Diameter | 0.09548165 |
| Laterality | 0.20571188 |
| Multifocality | 1.80447827 |
| Tumor Location | 0.33952437 |
| Tumor Echogenicity | 0 |
| Tumor Shape | 0 |
| Tumor Margin | 0 |
| Tumor Calcification | -0.06886087 |
| Tumor Length | 0.25568442 |
| Tumor Vascularity | 0 |
| RTE | -0.14447401 |
| Invasion | 0.58587383 |
| BRAFV600E Mutation | -0.07443478 |
| Maximum Diameter of Suspicious LN on US | 0.12254301 |
| Multiregionality of Suspicious LN on US | 0.50575162 |
| Shape of Suspicious LN on US | 0 |
| Corticomedullary Differentiation of Suspicious LN on US | 0 |
| Calcification of Suspicious LN on US | -0.01172792 |
| Blood Flow Signals of Suspicious LN on US | 0.0536924 |
| CT reported LN Status | 2.01836373 |
| Shape of LN on CT | 0 |
| Margin of LN on CT | 0 |
| Calcification of LN on CT | 0.31580158 |
| Enhanced Types of LN on CT | 0.95202826 |
| FT3 | -0.0262118 |
| FT4 | 0 |
| TSH | 0 |
| TRAb | 0 |
| TG | 0.00740536 |
| TBG | -0.0041508 |
| TGAb | -0.01502082 |
| TPOAb | -0.00053648 |
| CEA | 0 |
